# Audit on clozapine dose and plasma level correlation for patients with chronic treatment-resistant psychosis

**DOI:** 10.1192/bjo.2021.273

**Published:** 2021-06-18

**Authors:** Olivia Macnamara, John Lawton, Sudheer Lankappa

**Affiliations:** Nottinghamshire Healthcare NHS Foundation Trust

## Abstract

**Aims:**

Clozapine is associated with a risk of severe adverse events for which there are current monitoring systems are in place; however, there are no established regimens for monitoring of clozapine plasma levels. Recent Medicines and Healthcare products Regulatory Agency (MHRA) guidance advises clozapine levels should be monitored in certain clinical situations where toxicity may be suspected. This audit aimed to evaluate current practice of clozapine level monitoring within one Local Mental Health Team (LMHT).

**Method:**

Electronic (RiO) records of 41 patients (33 male, 8 female; aged from 27 to 76 years; mean age 45 years) registered to the ZTAS system within the Nottingham City Central LMHT were reviewed. 46% had been on clozapine for over 16 years. 73.3% of patients were within clusters 12 and 13; 25.4% of patients were in cluster 11, with one patient in cluster 8. Dates of clozapine plasma level tests for each patient between 2006 and 2020 were found on the electronic NoTIS system, along with clozapine, norclozapine and total clozapine levels. Concurrent clozapine dose and regimens were obtained from pharmacy records from 2018 onwards.

**Result:**

273 clozapine plasma levels were conducted between 2006 and 2020. The average interval between levels taken was 10 months, 2 weeks but had a wide range, the shortest interval being 2 days, the longest being 13 years. 88 levels taken were >600 ug/L, suggesting increased toxicity risk. 108 levels were <350 ug/L, suggesting possible sub-optimal dosing or non-compliance. Statistical tests on correlation coefficient, although statistically non-significant (R = 0.37), showed a positive trend between total clozapine dose and the plasma level between all 3 parameters (i.e. clozapine, norclozapine and total clozapine).

**Conclusion:**

There does not appear to be any routine plasma clozapine level monitoring throughout the LMHT with an average interval between tests of 10 months. There was a non-significant but positive trend between total daily dose of clozapine and clozapine level. 32% of clozapine levels returned were higher than the recommended level. We would recommend as suggested in the guidelines from MHRA, clozapine plasma levels should be monitored in certain clinical situations with increased toxicity risk. Trough levels should be taken with records of time of previous dose taken. Limitations of this study included a small sample size (41 patients) with data collection reliant on electronic systems. It was unclear if these results represent trough levels, making values difficult to interpret. Multifactorial impact on clozapine metabolism causes wide patient variability in plasma levels.

